# Self-neglect among older adults with disabilities in Liaoning Province during the COVID-19 outbreak: A cross-sectional study

**DOI:** 10.3389/fpsyg.2022.1072110

**Published:** 2022-12-22

**Authors:** Li Dong, Longfeng Sun

**Affiliations:** ^1^Department of Nursing, The Second People's Hospital of Yibin, Yibin, China; ^2^Department of Geriatrics, The First Hospital of China Medical University, Shenyang, China

**Keywords:** associated factors, prevalence, self-neglect, social support, depressive symptoms, disability elderly, monthly income

## Abstract

**Background:**

Self-neglect among older adults is a well-recognized public health issue. During the novel coronavirus disease 2019 (COVID-19), older adults with disabilities may be at increased risk for self-neglect. This study aimed to assess the prevalence of self-neglect and associated factors among older adults with disabilities in Liaoning Province during the COVID-19 outbreak.

**Methods:**

A cross-sectional study was conducted from January 2021 to April 2021. A convenience sample of 230 older adults with disabilities filled out questionnaires that collected data on socioeconomic and demographic characteristics alongside data using the Barthel Index (BI), the Elder Self-Neglect Assessment (ESNA), the Geriatric Depression Scale-Short Form (GDS-SF), the Multidimensional Scale of Perceived Social Support (MSPSS), and the Mini-Mental State Examination (MMSE).

**Results:**

The overall self-neglect rate was 86%. The study revealed that self-neglect correlates positively with the degree of disability (*p* < 0.001) and depressive symptoms (*p* < 0.001). In contrast, monthly income (*p* = 0.002) and perceived social support (*p* = 0.001) were negatively associated with self-neglect.

**Conclusion:**

During the COVID-19 outbreak, self-neglect has been a serious public health problem among older adults with disabilities. Lower monthly income, decreased physical function, depressive symptoms, and perceptions of poor social support contributed to self-neglect among older adults with disabilities.

## Introduction

In 2020, the novel coronavirus disease 2019 (COVID-19) broke out in China and soon became a global pandemic. Social isolation has been proposed as one of the main strategies to control it, which has caused many difficulties in people's daily lives. Self-neglect among older people is a well-recognized public health issue (Dong, 2017). According to the National Center on Elder Abuse in the United States, older adult's self-neglect is “an older adult's refusal or failure to provide himself/herself with essential self-care tasks, including keeping up personal hygiene, searching for medical assistance when necessary, and maintaining a socially accepted standard of health” (National Center on Elder Abuse, 2017). The prevalence of older adult self-neglect varies depending on the operational definition of the problem, the measurement instruments, and the study populations (Dong, 2017). In Korea, the prevalence of self-neglect was found to be 22.8% for older people who live alone (Lee and Kim, 2014). Ilhan et al. (2020) found that the prevalence of self-neglect was 16.8% among community-dwelling, self-sufficient older adults in Istanbul. A community study in the United States showed a 19.2% prevalence of self-neglect in individuals over 60 years (Dong et al., 2010).

There have been numerous efforts to identify factors associated with the risk of self-neglect. Recent studies showed that female gender, old age, having a lower education level, having decreased physical function, lack of social support, and participants diagnosed with depression are associated with a high risk of self-neglect (Dong et al., 2010; Lee and Kim, 2014; Dong and Simon, 2016; Ilhan et al., 2020). Self-neglect has been shown to be associated with an accelerated decline in physical condition, poorer health-related quality of life, and an increased risk of hospitalization (Dong et al., 2009; Dong and Simon, 2015; Zhao et al., 2017). To develop effective prevention programs, it is essential to improve our understanding of the factors influencing self-neglect. Since self-neglect significantly impacts older adult self-care, older adults with disabilities may be at higher risk of self-neglect. Besides, during the COVID-19 outbreak, older adults with disabilities are more likely to have mental health problems (Li et al., 2021), which may increase the risk of self-neglect.

The world has an aging population, leading to a rise in disability (WHO, 2015). Statistics show that, by 2020, the number of older adults with disabilities in China had reached 42 million (Zhang et al., 2020). This raises a growing concern about the maintenance and development of quality of life for older adults with disabilities. Older people with disabilities face challenges of comorbidities, poor social support, and inadequate economic support, which reduce their quality of life (Córdoba and Bagnato, 2021). These disadvantages may also put older adults with disabilities at increased risk for self-neglect (Dong, 2017). Previous studies found that older people with impaired physical function are at greater risk of self-neglect (Dong et al., 2009; Ilhan et al., 2020). Affected by the COVID-19 outbreak, this kind of situation may be even more serious.

Research on self-neglect, however, has mostly focused on self-sufficient older people (Lee and Kim, 2014; Ilhan et al., 2020). Despite their apparently greater risk of self-neglect, there is a lack of research regarding the prevalence of self-neglect among older adults with disabilities during the COVID-19 outbreak. Therefore, this study aimed to investigate the prevalence and associated factors of self-neglect among older adults with disabilities in Liaoning Province during the COVID-19 outbreak.

## Methods

### Design and setting

We adopted a cross-sectional descriptive correlational design, which was conducted in two communities in a district of Shenyang, China from January 2021 to April 2021. Participants were enrolled in the survey through convenience sampling. The inclusion criteria were older adults who: (1) met the disability criteria of the Barthel Index (score ≤95), (2) were aged 65 years and above, and (3) were able to communicate in Mandarin Chinese. The exclusion criteria were older adults who: (1) had impaired bilateral hearing or vision and (2) had a diagnosis of dementia confirmed by physicians.

### Research instruments

#### Sociodemographic characteristics questionnaire

Participants were asked to complete several sociodemographic items related to their gender, age, educational level, monthly income, marital status, smoking, drinking habits, place of residence, and the number of chronic diseases.

#### Barthel index (BI)

BI (Mahoney and Barthel, 1965) was used to assess participants' functional abilities. The scale includes ten items: feeding, grooming, bathing, dressing, bowel control, bladder control, toilet use, chair/bed transfers, ambulation, and stair-climbing. Total scores range from 0 to 100, with higher scores indicating better functional status. Participants were grouped into three categories according to their scores: mild disability (65–95), moderate disability (45–60), and severe disability (<40).

#### The scale of elderly self-neglect (SESN)

Self-neglect was measured by the 14-item instrument developed by Iris et al. (2010). The SESN was classified into five phenotypes: medical health and care, environmental sanitation and personal hygiene, mental health, safety, and social communication. Each question had four options: not occurring or no effect, mild effect, moderate effect, and severe effect. The total score is 42. A higher score indicates a higher level of self-neglect. The Chinese version has good reliability and validity, with a Cronbach's alpha of 0.92 (Zhao et al., 2017). The Cronbach's alpha coefficient for the scale in this sample was 0.87.

#### Geriatric depression scale-short form (GDS-SF)

The GDS-SF (Zimet et al., 1988) was used to measure the depressive symptoms of the participants. The GDS-SF consists of 15 items using a dichotomous response of “yes” or “no” (0 = no, 1 = yes). The total score ranges from 0 to 15. Participants with a GDS-SF score of ≥5 were classified as having depressive symptoms. The GDS-SF was validated in Chinese, and Cronbach's alpha was 0.83 (Nyunt et al., 2009). The Cronbach's alpha coefficient for the scale in this sample was 0.91.

#### Mini-mental state examination (MMSE)

The MMSE scale (Folstein et al., 1975) was used to assess the participants' cognitive function. The scores of the MMSE range from 0 to 30, and higher scores indicate better cognitive functions. Cognitive impairment was defined as a score of ≤17 for illiterate participants, a score of ≤20 for participants with primary school education, and a score of ≤24 for those with a junior high school degree or above. The validity and reliability of the Chinese version of the MMSE have been verified (Katzman et al., 1988).

#### Multidimensional scale of perceived social support (MSPSS)

Participants' perceptions of social support were measured using the MSPSS (Zimet et al., 1988). The MSPSS includes family support, support from friends, and support from significant others. Each item was rated on a seven-point Likert scale. The total score ranged from 12 to 84, with a higher score indicating a higher level of support perceived by the participant. Participants were grouped into three categories according to their scores: low support (12–36), intermediate support (37–60), and high support (61–84) (Jiang, 2001). The Cronbach's alpha coefficient for the scale in this sample was 0.90.

### Data collection procedure

All procedures in the study involving human participants were in accordance with the basic principles of the Declaration of Helsinki and were approved by the Ethics Committee of China Medical University (approved in 2020, no. 392). All participants provided written informed consent. All the participants underwent a comprehensive assessment conducted by two trained researchers.

### Data quality assurance

To ensure the quality of the data, we used properly designed data collection tools, and the questionnaire was pretested on 5% of the sample size to check whether the instrument was adequately understood. The data were collected through face-to-face interviews with participants. The researcher reviewed the collected data and checked for completeness at the end of each day.

### Statistical analysis

All statistical analyses were performed using IBM SPSS® (version 22.0). The Kolmogorov–Smirnov test was used to determine the normality of distributions. According to the nature of the data, descriptive statistics, such as frequencies, prevalence, and median (interquartile range), were presented. As the data on self-neglect had a non-normal distribution, the Kruskal–Wallis test or Mann–Whitney U-test was used to examine the differences in self-neglect among different demographic characteristics as appropriate. Multiple linear regression was performed to identify which factors were independent predictors of self-neglect. A variance inflation factor (VIF) value was used to evaluate multicollinearity. The results were considered significant when a p-value was <0.05.

## Results

### Characteristics of participants

A total of 230 older adults with disabilities were investigated in this study. The mean age of the study sample was 69.25 ± 7.27 years old. In terms of monthly income, 50% (*n* = 115) of the participants had a monthly income of less than ¥3,000 (approximately USD $464). Only 7.8% (*n* = 18) of those had a monthly income of more than ¥5,000 yuan (approximately USD $773). Regarding the degree of disability, 57.4% (*n* = 132) of participants were classified as having a mild disability, 22.6% (*n* = 52) as having a moderate disability, and 20% (*n* = 46) as having a severe disability. Table 1 presents detailed information on the characteristics of the participants. At the time of the survey, the prevalence of self-neglect among older adults with disabilities was found to be 86%.

**Table 1:** Characteristics of the participants.

**Variables**	***N* (Percentage)**	**Mean** **(SD)**	**Median** **(IQR)**
**Age**			
65–74	151 (65.6)		
75–84	54 (23.5)		
≥85	25 (10.9)		
**Gender**			
Men	147 (63.9)		
Women	83 (36.1)		
**Educational level**			
Primary school or below	69 (30.0)		
Middle school	81 (35.2)		
High school or above	80 (34.8)		
**Marital status**			
Married	184 (80.0)		
Divorced	12 (5.2)		
Separated	31 (13.5)		
Unmarried	3 (1.3)		
**Monthly income (RMB)**			
≤3000	115 (50.0)		
3000–5000	97 (42.2)		
≥5000	18 (7.8)		
**Number of chronic diseases**			
1	106 (46.1)		
2	101 (43.9)		
≥3	23 (10.0)		
**Place of residence**			
City	181 (78.7)		
Rural	49 (21.3)		
**Smoking**			
Yes	193 (83.9)		
No	37 (16.1)		
**Drinking**			
Yes	199 (86.5)		
No	31 (13.5)		
**Degree of disability**			
Mild disability	132 (57.4)		
Moderate disability	52 (22.6)		
Severe disability	46 (20.0)		
BI			65.00 (45.00,75.00)
SESN			9.00 (4.74,12.25)
MSPSS		52.16 (8.13)	
GDS-SF		4.07 (3.25)	
MMSE		24.08 (4.40)	

According to the exchange rate on July 4, 2022, 1RMB = 0.1495USD. BI, Barthel Index; SESN, Scale of Elderly Self-Neglect; MSPSS, Multidimensional Scale of Perceived Social Support; GDS-SF, Geriatric Depression Scale-Short Form; MMSE, and Mini-Mental State Examination.

### Differences in self-neglect among various characteristics

As presented in Table 2, older adults with disabilities who had higher levels of education and higher monthly income levels had lower levels of self-neglect (*p* = 0.048 and *p* < 0.001, respectively). There was a significant relationship between self-neglect and marital status (*p* = 0.009). The median score of self-neglect for those with severe disabilities was much higher than that for mild and moderate disabilities (*p* < 0.001). A lower level of perceived social support was significantly associated with higher levels of self-neglect (*p* < 0.001). Cognitive impairment and depressive symptoms were significantly associated with higher levels of self-neglect (*p* = 0.037 and *p* < 0.001, respectively). There were no significant differences in other characteristics.

**Table 2:** Self-neglect comparisons among different characteristics.

**Variables**	**Self–neglect**	**X^2^/Z**	***P*–value**
**Age**		5.48	0.139
65–74	7 (4–12)		
75–84	8 (5–14)		
≥85	10 (5–15)		
**Gender**		−0.21	0.826
Men	8 (4–12)		
Women	8 (4–14)		
**Educational level**		5.80	0.048
Primary school or below	10 (5–14.5)		
Middle school	8 (5–13.5)		
High school or above	6 (3–10)		
**Marital status**		11.51	0.009
Married	7 (4–12)		
Divorced	4.5 (3–8.25)		
Separated	11 (4–16)		
Unmarried	9 (3.75–11.25)		
**Monthly income (RMB)**		17.91	<0.001
≤3000	10 (5–14)		
3000–5000	6 (4–12)		
≥5000	4.5 (2.75–7.25)		
**Number of chronic diseases**		3.01	0.221
1	10 (5.75–12.5)		
2	6 (4–12)		
≥3	8 (4–12.25)		
**Place of residence**		0.94	0.331
City	7 (4–12)		
Rural	9 (4–15)		
**Smoking**		−1.67	0.094
Yes	6 (3–11.5)		
No	8 (4–12.5)		
**Drinking**		−0.95	0.338
Yes	6 (3.25–12)		
No	8 (4–12)		
**Degree of disability**		92.43	<0.001
Mild disability	5 (4–9)		
Moderate disability	8 (5–11)		
Severe disability	16 (12–20)		
**Depressive symptoms**		−6.10	<0.001
Yes	12 (10–16)		
No	6 (4–10)		
**Cognitive impairment**		−2.09	0.037
Yes	9.5 (5–16)		
No	7 (4–12)		
**Perceived social support**		20.53	<0.001
Low support	16 (9–21)		
Intermediate support	8 (5–12)		
High support	4 (3–6)		

### Predictors of self-neglect

Multiple linear regression analyses were used to determine the predictors that might contribute to self-neglect for older adults with disabilities. The total self-neglect score was the dependent variable. Educational level, monthly income, marital status, degree of disability, depressive symptoms, cognitive function, and perceived social support were the independent variables. The overall multiple regression model was statistically significant (adjusted R^2^ = 0.52, F = 31.95, *p* < 0.001), accounting for 52% of the variance in self-neglect scores.

Monthly income (β = −0.17, *p* = 0.002), the degree of disability (β = 0.52, *p* < 0.001), depressive symptoms (β = 0.18, *p* < 0.001), and perceived social support (β = −0.15, *p* = 0.001) were identified as significant predictors of self-neglect. Perceived social support and monthly income were negatively correlated with the self-neglect scores, while the degree of disability and depressive symptoms were positively correlated (Table 3). The results of the VIF (all less than 1.5) and collinearity tolerance (all greater than 0.7) suggest that the estimated βs are well supported.

**Table 3:** Predictive factors of self–neglect according to the multiple linear regression.

**Variables**	**B**	**β**	**T**	***p*–value**	**95% CI**
Educational level	0.27	0.04	0.74	0.460	−0.45, 1.01
Monthly income	−1.41	−0.17	−3.15	0.002	−2.30, −0.53
Degree of disability	3.69	0.52	10.07	0.000	2.97, 4.42
Depressive symptoms	2.51	0.18	3.74	0.000	1.19, 3.83
Cognitive function	0.67	0.04	0.95	0.340	−0.71, 2.07
Perceived social support	−2.07	−0.15	−3.26	0.001	−3.33, −0.82
Marital status 1^a^	−0.85	−0.04	−0.95	0.343	−2.61, 0.91
Marital status 2^b^	0.10	0.01	0.15	0.874	−1.18, 1.38
Marital status 3^c^	0.87	0.02	0.42	0.669	−3.13, 4.87

R^2^ = 0.52, F = 31.95, P < 0.001.

a 0 = Married, separated, or unmarried, 1 = divorced.

b 0 = Married, divorced, or unmarried, 1 = separated.

c 0 = Married, divorced, or separated, 1 = unmarried. CI, Confidence Interval.

## Discussion

Our study found that self-neglect was common among older adults with disabilities during the COVID-19 outbreak. Physical function impairment was the most significant and essential feature that distinguished them from other groups. It is well established that a decline in physical function leads to a decline in an individual's mobility and ability to self-care. Several prior studies found that older adults whose physical function declines have an increased risk of self-neglect (Dong et al., 2010; Lee and Kim, 2014; Ilhan et al., 2020). Older people with disabilities, therefore, are a vulnerable population in terms of self-neglect.

We examined the prevalence of self-neglect among older adults with disabilities in China. The prevalence of self-neglect in this study was 86%, which was higher than studies in Korea (22.8%), Istanbul (16.8%), and the United States (19.2%) (Dong et al., 2010; Lee and Kim, 2014; Ilhan et al., 2020). This may be due to a difference in our study population, which was made up of older participants with disabilities whose physical function decline made them more susceptible to self-neglect. Another reason may be variations in the tool used for measuring self-neglect. In Istanbul, for example, the study used the Istanbul Medical School Elder Self-Neglect questionnaire (IMSelf-neglect). The IMSelf-neglect scale, which mainly investigates whether older adults neglect their personal hygiene, health habits, and social functioning, differs from the measurement tool used in our study. Besides, social distancing and self-isolation measures during the COVID-19 outbreak imposed substantial burdens on older adults with disabilities, leading to an increased risk of self-neglect.

Examining sociodemographic characteristics, we found that marital status and education level were not associated with self-neglect, while monthly income had a strong negative association with self-neglect. The results of our study are similar to a cross-sectional study reported by Yu et al. (2019), which showed that older people with lower monthly incomes tended to have higher levels of self-neglect. This may be because older people with high monthly income levels have greater opportunities to access healthcare services and participate in more social activities, which may reduce the risk of self-neglect (Yu et al., 2019). However, this finding contrasted with the findings presented by Dong, 2016; who reported that monthly income had no significant association with self-neglect. Our study did not show differences in the risk of self-neglect based on the participant's gender or age, in contrast to the study by Abrams et al. (2002); in this study, it was observed that men and people of an older age tended to suffer self-neglect more frequently. Our study found that the number of chronic diseases was not associated with self-neglect. This finding is inconsistent with studies that indicated that increasing medical comorbidities were frequently seen in reported self-neglect cases among older adults (Reyes-Ortiz, 2001; Smith et al., 2006). Such a research discrepancy may be due to the heterogeneity of the study design and the disabled and elderly populations. Further studies are needed to better examine the associations between sociodemographic variables and self-neglect.

We found that a severe degree of disability was associated with higher levels of self-neglect than mild and moderate disabilities. A previous study reported that a decline in physical function was associated with an increased risk of greater severity of self-neglect (Dong et al., 2010). Our participants who needed help with activities of daily living were at a higher risk than others, similar to the findings of another study that indicated poor physical health appeared to be an independent predictor of self-neglect (Dong et al., 2009). Physical function impairment was also one of the common elements that contributed to self-neglect, as illustrated in the model of self-neglect developed by Dyer et al. (2007). According to Dyer et al. (2007), impairment in the activities of daily living is central to worsening vulnerability in the self-neglect syndrome.

Depressive symptoms were found to be significantly correlated with self-neglect in older adults with disabilities. During the COVID-19 outbreak, older adults with disabilities were afraid of being infected by the virus. They could not participate in normal outdoor activities, which was more likely to cause symptoms of depression (Li et al., 2021). The results of our study showed that the more depressive symptoms suffered by older adults with disabilities, the higher their levels of self-neglect. Another study on older people living alone revealed a positive correlation between depressive symptoms and self-neglect (Lee and Kim, 2014). Older people with depressive symptoms tend to have a lower life expectancy, a loss of interest or pleasure in activities, and a sense of hopelessness (Mulat et al., 2021), all of which may increase their risk for self-neglect. Predictably, higher levels of depression also significantly predict higher suicidal ideation among older adults with disabilities (Lund et al., 2016). In turn, suicidal ideation is associated with an increased risk of self-neglect (Dong et al., 2017).

Another finding of our study was that perceived social support was negatively correlated with self-neglect in that people with lower levels of perceived social support were at significantly higher risk of self-neglect. Owing to social isolation, older adults with disabilities receive less social support during the pandemic, which may increase the risk of self-neglect. Higher levels of social support lead to lower levels of psychological distress, and social support can be used to maintain a stable psychological state (Khatiwada et al., 2021). Perceptions of social support also significantly contribute to older people's life satisfaction and self-esteem (Farriol-Baroni et al., 2021). Our results suggest that higher perceived social support levels might play a protective role against self-neglect. Therefore, support from family, friends, and significant others helps prevent self-neglect.

Compared with normal cognition, our study found that cognitive impairment was associated with higher levels of self-neglect in the univariate analysis, although it was not statistically significant in the multilinear regression model. Some previous studies reported that cognitive impairment tended to be a risk factor for self-neglect (Dong and Simon, 2016), while Lee and Kim (2014) observed a negative association between cognitive impairment and self-neglect. The inconsistency suggests a complex relationship between cognitive impairment and self-neglect among older adults. Thus, further studies are needed to explore the association between cognitive impairment and self-neglect.

Our study also found that most respondents did not consider their behaviors self-neglect. This may be due to inadequate sensitization regarding the problem. Therefore, education, perhaps on television or in newspaper articles, is vital for helping older people deepen their understanding (Zhao et al., 2017).

Our study has several limitations. First, the incidence of self-neglect in older adults with disabilities was significantly different from the results of previous studies, which an overestimation of the SESN and incorrect cut-off values may cause. Therefore, future studies need to further verify the reliability and validity of the scale to accurately assess self-neglect among older adults with disabilities. Second, only correlations rather than causal relationships can be established, as our results were based on a cross-sectional study. Further longitudinal studies are necessary to clarify the relationships between these risk factors and self-neglect. Third, all participants were from two communities in a district in Shenyang, China, making the findings less generalizable to other regions of the country. Fourth, variables such as alcohol, educational status, and cigarette smoking are sensitive issues and may have caused a social desirability bias. Finally, as this study used a convenience sample, there may be selection bias in the results. For example, the majority of this sample were men aged 65–74. In future studies, random sampling should be selected to ensure the representativeness of the samples.

## Conclusions

Our study found that self-neglect was common among older adults with disabilities during the COVID-19 outbreak. We found that depressive symptoms, a higher degree of disability, lower levels of perceived social support, and a lower monthly income were significantly associated with high levels of self-neglect among older adults with disabilities in China.

Given the global and ongoing increase in self-neglect among older people, these findings have important implications for designing appropriate interventions for preventing and managing contributing factors to self-neglect among older adults with disabilities.

## Data availability statement

The original contributions presented in the study are included in the article/supplementary material, further inquiries can be directed to the corresponding author.

## Ethics statement

Written informed consent was obtained from the individual(s) for the publication of any potentially identifiable images or data included in this article.

## Author contributions

LD and LS conceived and designed the study, performed the data analysis, and wrote the manuscript. All authors read and approved the final manuscript.
